# Correlating grain yield with irrigation in a spatio-temporal context on the North China Plain

**DOI:** 10.1016/j.heliyon.2024.e32745

**Published:** 2024-06-14

**Authors:** Yulian Gao, Yaojie Yue, Wuqiong Yang

**Affiliations:** Key Laboratory of Environmental Change and Natural Disasters of Chinese Ministry of Education, Faculty of Geographical Science, Beijing Normal University, Beijing, 100875, China

**Keywords:** Grain yield, Irrigation, Spatio-temporal correlation, Irrigation strategies, North China Plain

## Abstract

Irrigation plays a crucial role in enhancing agricultural productivity. The spatiotemporal variability and correlation between historical irrigation and grain yield not only illuminate existing challenges in irrigation, but also offer valuable insights for formulating effective irrigation strategies, which have been previously overlooked. Taking the North China Plain (NCP) as a case study, this study aims to elucidate regional divergence patterns and the dynamic evolution of the spatiotemporal relationship between grain yield and irrigation through time series analysis, GIS spatial analysis, and geographically weighted regression (GWR). The findings reveal that grain yields are higher in the northern regions of NCP compared to the southern regions, with significant variations among prefecture-level cities; maize yields slightly surpass wheat yields. Moreover, there has been a noticeable decrease in irrigation across approximately 49 % of the areas since 2004. Spatial autocorrelation analysis indicates clear spatial aggregation for both grains yields and irrigation. The coupled correlation between wheat yield and effective irrigation has shown a slight increase from 1990 to 2015, while that of maize has significantly decreased. The positive impact of irrigation on grain yield has nearly vanished since 2002. It is recommended to implement sprinkler irrigation in low-yield, low-irrigation areas in the south; deficit irrigation and water-saving technologies may benefit regions with medium yield and negative correlation with irrigation in central parts; maintaining current irrigation strategies is suggested for high-yield and high-irrigation regions. Additionally, relying solely on irrigation to boost yields is unsustainable; it is critical to adopt a combination of agricultural management practices along with planting high water-utilization efficient crop varieties. This study underscores the significance of developing rational irrigational strategies based on a comprehensive understanding of the intricate relationship between irrigation and grain yields-ensuring food security while sustaining agricultural water utilization.

## 1 Introduction

Irrigation, as an important way of securing agricultural production, constantly plays an important role in the context of continuous population growth, increasing demand for food, and limited expansion of cultivated land [[1], [2], [3]]. However, excessive irrigation causes many negative impacts on agricultural systems and even the regional water and soil resources [[4], [5], [6], [7]], such as a decrease in the runoff and underground water resources [8], accelerated desertification [9] and secondary salinization of soil [10]. While inadequate irrigation usually leads to unstable agricultural production. Therefore, this highlights the importance of understanding the geographical correlation between irrigation and agricultural production in different geographical units [11,12], which is of great significance for improving irrigation strategies. Given the pivotal role of irrigation in stabilizing global agricultural production, there remains a pressing need for a more comprehensive investigation to propose evidence-based irrigation strategies grounded in a thorough understanding of the geographical correlation between irrigation and grain yield. More importantly, such irrigation strategies have global significance for conserving natural resources while increasing food production to meet the needs of a growing population.

Among the various methodological to quantify the relationship between grain yield and irrigation, field experiments, quantitative statistics and the multi-model combination analysis have been widely applied. By setting different irrigation levels, the mechanisms of irrigation effects on grain yield have been revealed quantitatively based on a set of field experiments [[13], [14], [15], [16], [17]]. For example, many studies by comparison experiments with different irrigation levels, showed that the irrigation water utilization efficiency decreased as the amount of the irrigation water increased [18]. In other words, proper irrigation is effective in increasing grain yield, while over irrigation reduces the efficiency of yield increase [14,19]. Though quantitative relationship between grain yield and irrigation can be obtained by field experiments. However, such experiments based on short-term period and are usually conducted in specific areas by strictly controlled conditions, which can hardly apply to reveal the relationships between yields and irrigation over the long-term period and large-scale areas.

Quantitative statistical models are able to compensate the deficiency of field experiments, which have been widely used to mine the quantitative relationship between grain yield and irrigation over large scale areas [4,20]. This type of approach is based on long time-series data and the quantitative relationship between grain yield and irrigation is generally revealed by regression analysis, Cobb-Douglas production function, and Bayesian model [3,[21], [22], [23]]. For example, the Bayesian model was utilized to quantify the contribution of irrigation to global crop yields, approximately 80–126 million ha of modern rainfed wheat and maize cropland worldwide do not have access to sufficient discharge to meet irrigation demand [3]. However, statistical methods are significantly impacted by data quality. Moreover, and most importantly, statistical methods do not have the capacity for spatial analysis, i.e., regional differences in the quantitative relationship between grain yield and irrigation can hardly be revealed by those methods.

With the support of the geographic information system (GIS), the quantitative relationship between grain yield and irrigation was revealed by the multi-modeling approach, including statistical models, spatial analysis models, gray correlation models, and crop models [5,[24], [25], [26]]. For example, combined with the GIS and APSIM model, Chen et al. [27] found that grain yield was higher in the north under full irrigation in the North China Plain (NCP) from 1961 to 2005. The GIS-based multi-model approach is currently widely used in revealing the quantitative relationship between grain yield and irrigation. Compared with the field experiments and statistics, GIS-based multi-models have the potential to reveal regional differences, especially with the support of large-scale regional and long time-series data. However, prior studies using multi-model combination approaches have rarely considered the spatial and temporal variability in the quantitative relationship between grain yield and irrigation. Which stems from the significant temporal differences in crop growth and irrigation conditions in different regions.

In China, the NCP is one of the most important agricultural regions, suffering severe groundwater over-consumed and contributing to over one-third of the national wheat and maize production [[27], [28], [29], [30], [31]]. The agricultural production of NCP heavily relies on irrigation [32], which is one of the most fundamental field practices, strongly influenced by climate change, and is a critical guarantee for determining regional food production [20,25,26,33,34]. A large amount of research has analyzed the impact on food production from the perspective of improved irrigation technologies and irrigation strategies [16,29,[35], [36], [37]]. However, rational irrigation strategies arising from historical spatial and temporal coupling characteristics of irrigation and grain production have rarely been explored in this high-intensity irrigated agricultural area.

In summary, the present study aims to elucidate the quantitative relationship between grain yield and irrigation across various temporal and spatial scales by adopting a method that is capable of interpreting the spatial heterogeneity and correlations of irrigation and grain yields. Subsequently, tailored irrigation strategies will be proposed based on regional variations in grain yield and spatio-temporal patterns of irrigation. Using the North China Plain (NCP) as a case study, this research analyzes the spatio-temporal distribution pattern of grain production and irrigation from 1990 to 2015, evaluates the spatio-temporal correlation characteristics of grain yield and irrigation, and discusses the coupling pattern of grain yield and irrigation along with its dynamic evolution characteristics.

## 2 Method and data

### 2.1 Study area description

**Fig. 1:**
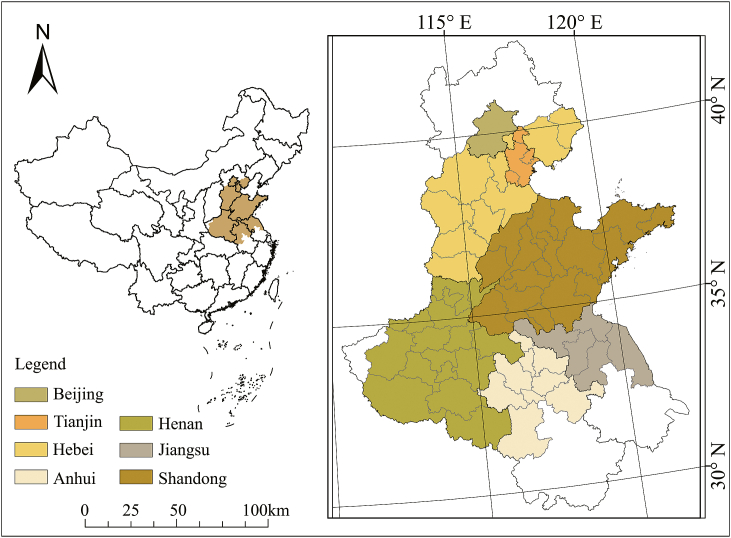
Location and extent of the North China Plain.

The NCP including 55 prefecture-level cities consisting of seven provinces, including Beijing, Tianjin, Hebei Province, Shandong Province, Henan Province, Anhui Province, and Jiangsu Province, covering an area of approximately 560,000 km^2^ (Fig. 1). As the most important grain production area in China [38], the NCP produces approximately 2/3 of the national total wheat yield [30] and plays an critical role in guaranteeing national food security. However, most of the NCP is a humid/semi-humid zone and is one of the severe water-scarce regions in China. Its annual average precipitation was 734.9 mm [39] which cannot meet the requirement of about 900 mm for wheat and maize growth, the two main crops in this region. The conflicts between irrigation water demand and water supply in most places in the NCP are very prominent [40].

### 2.2 Data sources

The agricultural regionalization (1988 version) and the administrative division of China (2015 version) were downloaded from the Resource and Environment Science and Data Center (http://www.resdc.cn). The grain yield and irrigation data were obtained from the online database of China National Knowledge Network (CNKI, https://data.cnki.net/). The original data was reported by various prefecture-level cities in China, and subsequently reviewed and published by the Ministry of Natural Resources and the Ministry of Agriculture and Rural Affairs, ensuring its reliability. CNKI is widely recognized as one of the largest and most authoritative digital repositories in China, with a rigorous quality control and error checking mechanism implemented during the digitization process of grain yield and irrigation data. Therefore, the sources and quality of the grain yield and irrigation data used in this study are deemed to be reliable.

### 2.3 Basic idea

To formulate reasonable regional agricultural irrigation strategies, the key depends on fully understanding the spatial and temporal variability of crop yields and irrigation at historical stages, and seeking clues from the characteristics of the coupled relationship between them [27,28,30]. Irrigation affects regional soil moisture and temperature, often interacting with crop growth, development, and crop yields [20,41,42]. Therefore, we will explore the characteristics of spatio-temporal relationships in terms of the respective change features of crop yields and irrigation, and the spatio-temporal relationships and interactions between crop yields and irrigation.

We achieve the research objectives through an analytical approach that combines spatio-temporal distribution with spatial statistical modeling. Firstly, a long-time series of yield and irrigation databases in the NCP is used to analyze the dynamics of crop yield and irrigation (Section 2.4.1 and Section 2.4.2). Second, the spatial patterns of grain yield and irrigation were revealed through GIS spatial analysis and global spatial autocorrelation analysis (Section 2.4.3). Finally, the temporal and spatial couple relationship between the yield and irrigation is obtained through the Geographically Weighted Regression (GWR) model (Section 2.4.4).

### 2.4 Methods

#### 2.4.1 Assessing the patterns of yields

The yield anomaly index (YAI) is an indicator used to assess the degree of deviation between crop yield and its long-term average yield [43]. The calculation formula as follows:

(1) $$\text{YAI}=\left[\frac{Y‐\mu }{\sigma }\right]\times 100\%$$

where *Y* is the annual crop yield, μ is the long-term average crop yield, and σ is the standard deviation of *Y*. The positive YAI values indicate that the actual yields exceed the average, while negative values suggest that the yields fall below the average. And a value of 0 indicates that the actual yields are equal to the average.

The crop yields change patterns are classified into six types: increasing slowly, increasing moderately, increasing rapidly, stagnated, never improved and collapsed growth [44].

#### 2.4.2 Assessment of irrigation

The Effective Irrigated Area Index (AEII) is a metric used to gauge the efficiency of irrigation systems. The AEII was described as:

(2) $${\text{AEII}}_{it}=\frac{{\text{AEI}}_{it}\times 1000}{A_{i\text{tw}}+A_{i\text{tm}}},i=1,2,\ldots ,n$$

where *AEII*_*it*_ represents the AEII of the *i*_th_ city in year t, *AEI*_*it*_ represents the effective irrigation area (1000 ha) of the *i*_th_ city in year t, *A*_*itw*_ represents the wheat sowing area (hectares) of the *i*_th_ city in year t, *A*_*itm*_ represents the maize sowing area (hectares) of the *i*_th_ city in year *t*, and *n* represents the quantities of cities in the NCP.

#### 2.4.3 Global spatial autocorrelation

In the present study, the global Moran's I index of global spatial autocorrelation [45] was adopted to determine the overall distribution and clustering of a certain attribute value at a spatial scale [46]. The index depicted as:

(3) $$I=\frac{n\sum_{i=1}^{n}\sum_{j=1}^{n}W_{ij}\left(X_{i}-\bar{X}\right)\left(X_{j}-\bar{X}\right)}{\sum_{i=1}^{n}\sum_{j=1}^{n}W_{ij}\sum_{i=1}^{n}{\left(X_{i}-\bar{X}\right)}^{2}}$$

where *W*_*ij*_ is the weighted function of space, and the numerical value is 1 when two areas are adjacent and 0 for nonadjacent; *X*_*i*_, *X*_*j*_ is the yield of the city units *i* and *j*, respectively; $\bar{X}$_and_ *X* are the mean values; and *n* is the total number of cities.

The significance of Moran's I index is usually verified with the standardized statistic *Z*:

(4) $$Z\left(I\right)=\frac{\;I‐E\left(I\right)}{\sqrt{\text{VAR}\left(I\right)}}$$

where E(I) is the mathematical expectation under the spatial random distribution hypothesis, and VAR(I) is the variance.

#### 2.4.4 Geographically weighted regression

The geographically weighted regression (GWR) model can reflect the spatial non-stationary of the relationship between elements in different spaces and can be used to calculate the relationship between different variables at different spatial positions [47]. The formula is as follows:

(5) $$y_{i}=\beta_{0}\left(u_{i},v_{i}\right)+\beta \left(u_{i},v_{i}\right)x_{i}+\varepsilon_{i},i=1,2,\ldots ,n$$

where $\left(u_{i},v_{i}\right)$ is the coordinate of the regional center of mass for the *i*_th_ city, $\beta \left(u_{i},v_{i}\right)$ is the regression coefficient of AEII for the *i*_th_ city, $\varepsilon_{i}$ is the random error of the *i*_th_ city, and *n* is the quantities of cities.

(6) $$\beta \left(u_{i},v_{i}\right)={\left(X^{T}W\left(u_{i},v_{i}\right)X\right)}^{‐1}X^{T}W\left(u_{i},v_{i}\right)Y$$

where *X* and *Y* represent the yield anomaly index matrix and the AEII matrix of each city, respectively. $\left(u_{i},v_{i}\right)$ is the spatial weighted matrix of the spatial coordinate point $W\left(u_{i},v_{i}\right)$ of each city.

When $\beta \left(u_{i},v_{i}\right)$ is positive, irrigation is positively correlated with grain yield. On the contrary, irrigation is negatively correlated with grain yield.

## 3 Results and discussion

### 3.1 Dynamics of crop yield

**Fig. 2:**
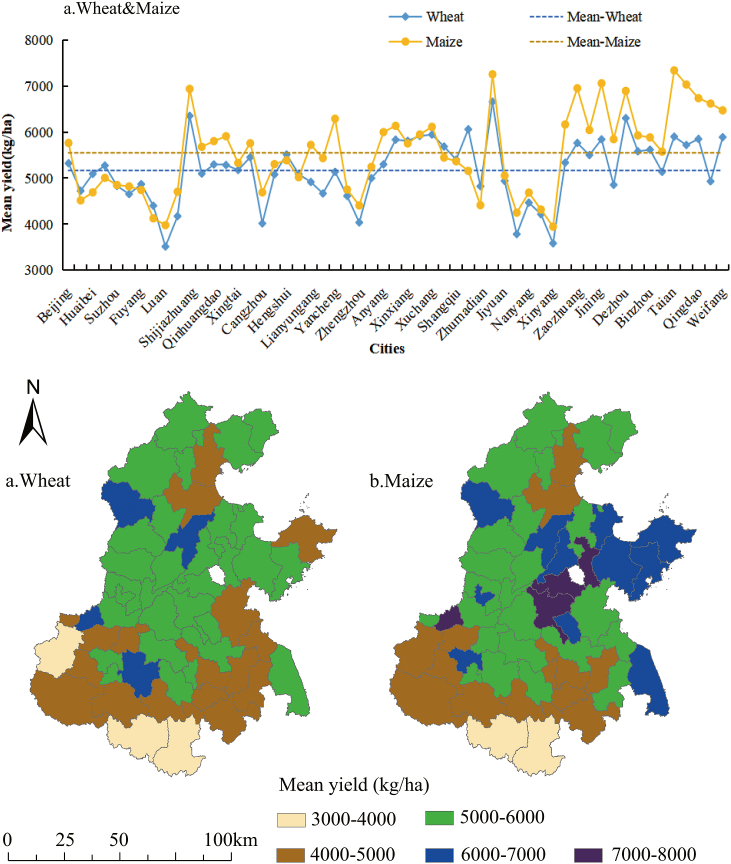
Spatial yield variation of wheat and maize in the North China Plain.

The results of crop yield showed that the yields of winter wheat and maize ranged from 4000 to 8000 kg/ha in approximately 90 % of the NCP, and maize yields were slightly higher than those of winter wheat (Fig. 2a). The spatial patterns of average grain yield of winter wheat in the NCP from 1990 to 2015 (Fig. 2b) show that high in the north and low in the south. Among them, cities with the average winter wheat yield higher than 6000 kg/ha accounting for 7.3 %, while winter wheat yield lower than 4000 kg/ha accounting for 5.5 % (Fig. 2b). The spatial pattern of maize yield is lower in the south, higher in the middle and moderate in the north (Fig. 2c). Cities with the average maize yield higher than 7000 kg/ha and 4000 kg/ha accounting for 5.5 %, 3.6 %, respectively. The above results indicate that high crop production is more prominently distributed in the southern part of the NCP, whereas low production is primarily concentrated in the northern part.

Similar to a prior study reported, a warmer climate in the southern NCP shortens the crop growth season due to weaker solar radiation, with consequent higher wheat and maize yields in the north and lower in the south [38,48]. This indicates that the spatial variability of wheat and maize yields in the NCP is significant [49]. Moreover, the total winter wheat production in China accounts for about one-sixth of world production [50]. As a major grain-producing region of China, the NCP produced approximately 68.55 % of the nation's wheat and 22.89 % of its maize [[51], [52], [53]]. Therefore, with population growth and increasing food demand, promoting grain production in the NCP is undoubtedly crucial to ensuring the food security of China in the context of global warming.

**Fig. 3:**
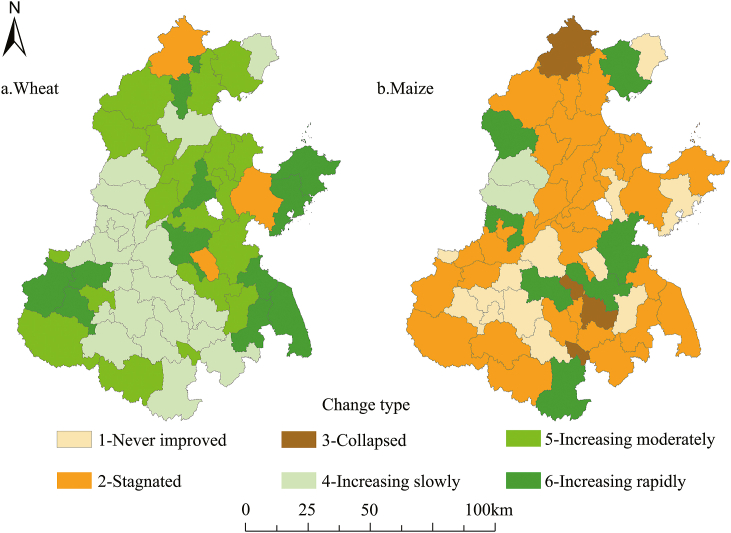
Yield change type in the North China Plain.

The changing patterns of crop yield show that wheat yields have continued to increase in 95 % of the cities (Fig. 3a), while maize yields have increased only slightly in 16.3 % of the cities (Fig. 3b). The proportion of low, medium and high growth patterns of winter wheat yields are 20 %, 34.5 % and 40 %, respectively. Among these, yields stagnated pattern accounting for 5 %. While another prior study reported a 61.1 % increase in wheat production in the NCP from 1981 to 2008 [44]. The increase in wheat yield may benefit from the application of fertilizer and pesticides [[54], [55], [56]] and the improvement of crop varieties [27,57], which increases the wheat yield in the NCP by about 12.2%–22.6 % [58].

On the contrary, the change pattern of maize yield is mainly stagnation and with low growth, while the areas with high growth patterns only accounted for only about 3.6 % (Fig. 3b). Moreover, the spatial distribution of low-growth cities is the widest, accounting for 12.7 %. Cities with the pattern of 'yields never improved', 'yields stagnated' and 'yields collapsed' accounted for 23.6 %, 54.5 % and 5.5 %, respectively. This finding is consistent with previous research reporting that the maize yield in 87.8 % of the counties in the NCP tended to stagnate or even decrease [28,59,60].

The spatial autocorrelation analysis of grain yield from 1990 to 2015 revealed obvious patterns of spatial aggregation within the NCP (Table 1). Specifically, the spatial clustering characteristics of winter wheat yield were stronger before 2004, with the highest clustering levels in 1991 and 1998. While the spatial clustering characteristics of maize yield was enhanced fluctuation, with the most significant clustering observed in 2003.

**Table 1:** Moran's I index of the yield for the North China Plain.

Year	Wheat		Maize	
	Moran's I	Z	Moran's I	Z
1990	0.33	3.22^c^	0.21	2.35^b^
1991	0.62	6.72^c^	0.37	4.19^c^
1992	0.27	3.05^c^	0.30	3.28^c^
1993	0.25	2.97^c^	0.20	2.28^b^
1994	0.42	4.62^c^	0.53	5.66^c^
1995	0.47	5.10^c^	0.30	3.40^c^
1996	0.21	2.36^b^	0.40	4.35^c^
1997	0.10	1.29	0.14	1.70^a^
1998	0.62	6.69^c^	0.34	3.76^c^
1999	0.14	1.68^a^	0.30	3.38^c^
2000	0.36	3.94^c^	0.34	3.78^c^
2001	0.24	2.69^c^	0.28	3.12^c^
2002	0.20	2.24^b^	0.13	1.60
2003	0.48	5.25^c^	0.74	7.83^c^
2004	0.16	1.81^a^	0.33	3.63^c^
2005	0.21	2.41^b^	0.49	5.30^c^
2006	0.20	2.31^b^	0.30	3.32^c^
2007	0.22	2.54^b^	0.55	5.90^c^
2008	0.19	2.16^b^	0.47	5.05^c^
2009	0.12	1.44	0.43	4.76^c^
2010	0.20	2.31^b^	0.44	4.90^c^
2011	0.24	2.72^c^	0.50	5.51^c^
2012	0.24	2.64^c^	0.60	6.52^c^
2013	0.24	2.69^c^	0.56	6.11^c^
2014	0.26	2.94^c^	0.61	6.52^c^
2015	0.28	3.12^c^	0.49	5.28^c^

Note: Statistical significance t a, b, c.

a p < 0.1.

b p < 0.05.

c p < 0.01.

### 3.2 Changes in effective irrigation

**Fig. 4:**
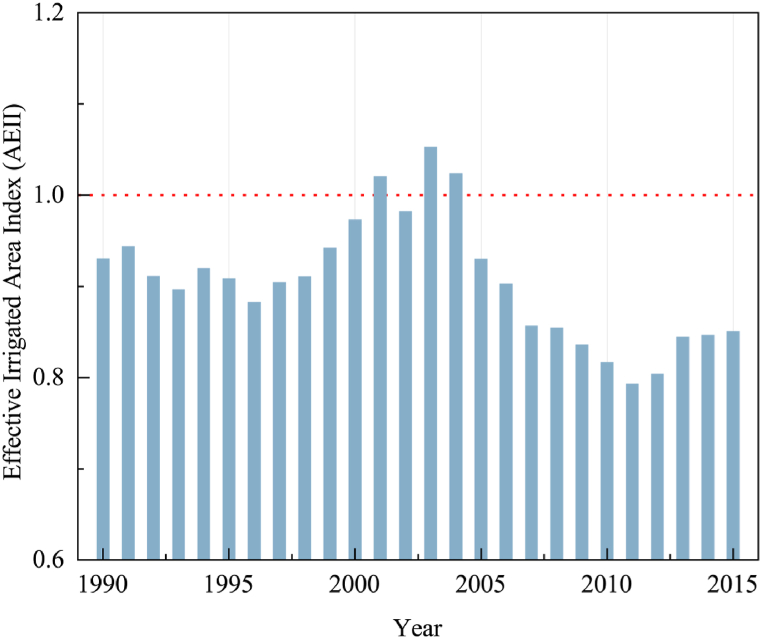
Average effective irrigated area index in the North China Plain from 1990 to 2015.

The average AEII in the NCP from 1990 to 2015 is shown in Fig. 4. AEII generally shows a trend of increasing and then decreasing in about 49 % of the areas, with peaks in AEII concentrated in 2001–2004. It is noteworthy that AEII exceeded 1 in 2001, 2003, and 2004, suggesting that the area effectively irrigated exceeded the area cultivated. This may imply that irrigation water had a significant surplus application in these three years. Moreover, the effective irrigated area in the NCP experienced a significant decline since 2004, with effective irrigation levels in 2010–2015 even lower than in 1990.

**Fig. 5:**
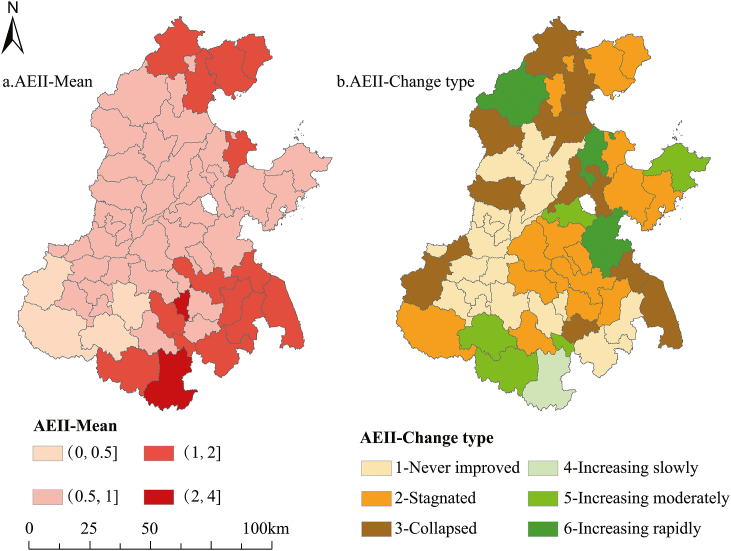
Mean AEII (a) in the North China Plain and change type (b).

The spatial patterns of AEII in the NCP revealed that effective irrigated areas remained at relatively low levels in over 70 % of the NCP, primarily concentrated in the central and western areas (Fig. 5a). Moreover, the AEII change type patterns suggesting that over 81 % of the areas experienced a irrigation intensity decline, only 16 % of the areas demonstrating a sustained increase in irrigation intensity (Fig. 5b).

Such a decline in effective irrigation areas of the NCP may be attributed to the serious water resource challenges. The NCP heavily reliant on groundwater resources for irrigated agriculture, stands as one of the four global regions facing severe groundwater overdrafts [40]. In recent years, with the development of urbanization and industrialization, the rapid development of the social economy has compressed the agricultural water supply [6]. The per capita water consumption in the NCP increased to 0.20 m^3^ in 2000 from 0.03 m^3^ in 1959. On the other hand, the exacerbation of climate aridification has led to an increase in the severity of drought in the NCP in recent years [39], resulting in declining precipitation and insufficient water for irrigation.

The spatial autocorrelation analysis of effective irrigation from 1990 to 2015 revealed obvious patterns of spatial aggregation within the NCP (Table 2). Moreover, Moran's index fluctuation increased, which means that the spatial aggregation of irrigation became more pronounced. The highest level of spatial aggregation of irrigation was observed in 1999. Whereas, in the years of high values of AEII, i.e., 2001, 2003, and 2004, the irrigation aggregation characteristics were lower than the multi-year average. This suggests that the NCP significantly increased irrigation in these years to promote increased food production, while a surplus of irrigation occurred.

**Table 2:** Moran's I index of the AEII for the North China Plain.

Effective Irrigated Area Index		
Year	Moran's I	Z
1990	0.33	3.22^c^
1991	0.62	6.72^c^
1992	0.27	3.05^c^
1993	0.25	2.97^c^
1994	0.42	4.62^c^
1995	0.47	5.10^c^
1996	0.21	2.36^b^
1997	0.10	1.29
1998	0.62	6.69^c^
1999	0.14	1.68^a^
2000	0.36	3.94^c^
2001	0.24	2.69^c^
2002	0.20	2.24^b^
2003	0.48	5.25^c^
2004	0.16	1.81^a^
2005	0.21	2.41^b^
2006	0.20	2.31^b^
2007	0.22	2.54^b^
2008	0.19	2.16^b^
2009	0.12	1.44
2010	0.20	2.31^b^
2011	0.24	2.72^c^
2012	0.24	2.64^c^
2013	0.24	2.69^c^
2014	0.26	2.94^c^
2015	0.28	3.12^c^

Note: Statistical significance at a, b, c.

a p < 0.1.

b p < 0.05.

c p < 0.01.

### 3.3 Coupling characteristics of yield and irrigation

**Fig. 6:**
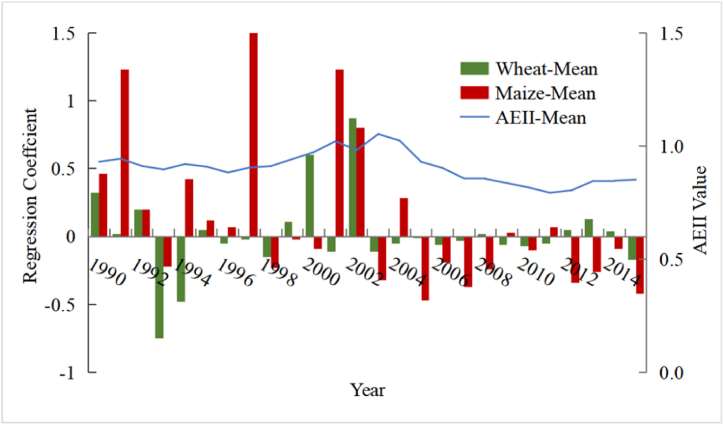
Temporal evolution of grain yield in relation to irrigation intensity.

The average regression coefficient of grain yield and irrigation in the NCP fluctuated up to the maximum value in 2002 (coefficient = 0.84), and then tended to decrease below than 0 since2003 (Fig. 6). Among them, the coupled correlation between wheat yield and effective irrigation increased slightly between 1990 and 2015, while that of maize decreased significantly. Irrigation had a significant yield-enhancing effect on wheat before 2001. However, the positive effect of irrigation on the increasing wheat yield has gradually disappeared since 2002. Moreover, the relationship between maize yield and irrigation displaying a significant increase and decrease between 1990 and 2003, while the degree of fluctuation decreased significantly after 2003 until it was basically negatively correlated (Fig. 6).

**Fig. 7:**
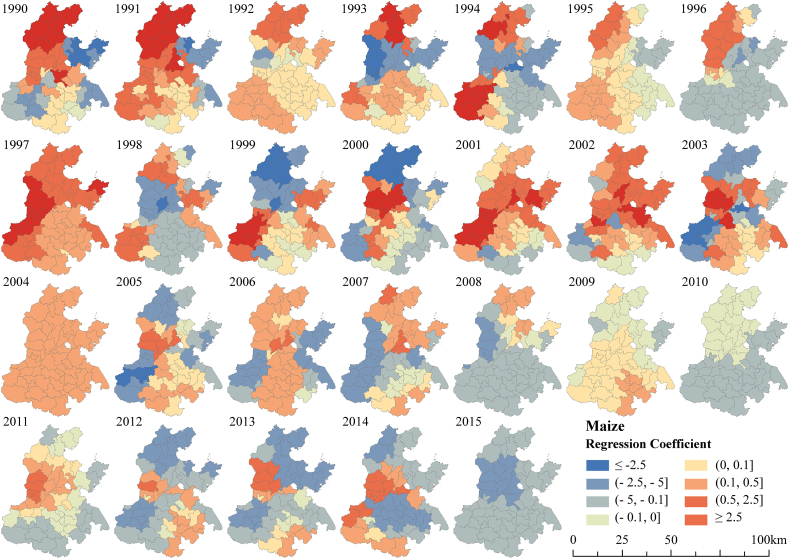
Spatial correlation patterns of maize yield and irrigation.

**Fig. 8:**
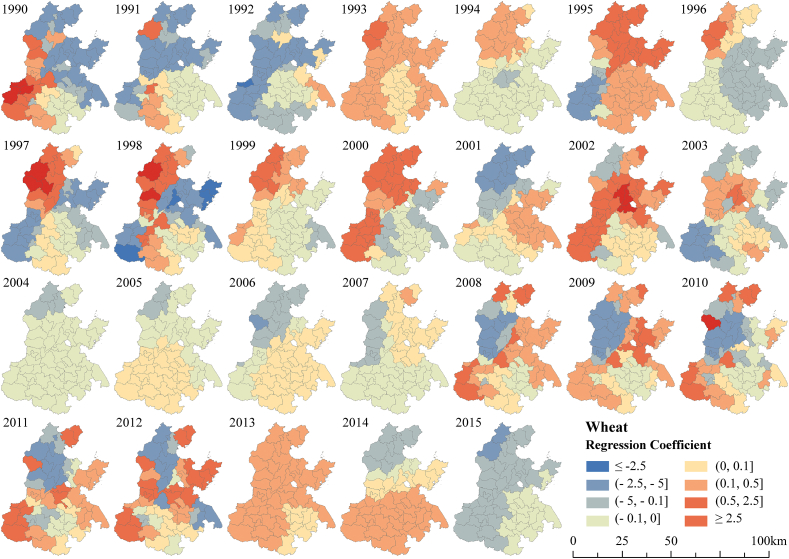
Spatial correlation patterns of winter wheat yield and irrigation.

The spatial distribution of regression coefficients between grain yields and irrigation were high in the north and low in the south (Fig. 7- 8). Similarly, under the full irrigation scenario, Chen et al. [27] found that grain yields were mostly higher in northern than in central and southern parts of NCP. The spatial correlation between maize yields and irrigation showed fluctuating changes of increasing and then decreasing (Fig. 7). The correlation between medium and low yielding areas with irrigation is generally low, indicating that irrigation has a weak effect on yield increase in these areas. The correlation between high yielding areas and irrigation first increased and then gradually decreased after 2007. This implies that the role of irrigation in increasing maize production in high-yielding areas has also passed its peak.

For winter wheat, the spatial correlation between yield and irrigation decreased first and then increased (Fig. 8), and the correlation was weaker than that for maize. Wheat yields in low-yield regions were mildly positively correlated with irrigation, suggesting that irrigation contributed little to increasing yields. In addition, the correlation between wheat yield and irrigation in the middle and high production areas first increased and then decreased, indicating that the positive effect of irrigation on yield is diminishing.

Irrigation had both positive and negative effects on maize yields in the NCP during 1990–2015, and regional differences varied significantly. However, the enhanced effect of irrigation on wheat yields gradually weakened, and the effect of irrigation on maize yield showed a decreasing trend since 2002. Some studies have found that the unit input consumption of irrigation water in terms of yield improvement has been decreasing [34]. This may be explained by the continuous decline in irrigation while the climate tends to warm and dry [61,62], which in turn leads to a gradual disappearance of the yield-increasing effect of irrigation on grain production.

### 3.4 Irrigation strategies based on the correlation between yields and irrigation

In the context of climate change, ensuring stable and increased yields in the NCP through increased irrigation is extremely challenging. Climate change affects grain production directly in terms of temperature [63], precipitation [54], and solar radiation [64], but also indirectly by affecting irrigation capacity. At the same time, several previous studies have found that the mismatch between agricultural practices and seasonal climate would exacerbate yield gaps and reduce resource use efficiency [65]. However, irrigation is typically used to mitigate drought risk, not only face a shortage of water for irrigation [66], but also can in turn exacerbate the intensity of heat waves [29]. Therefore, in the context of low availability of irrigation water, developing appropriate irrigation strategies incorporating finer regional geographic units that combine multiple field management practices with rational irrigation water allocation is critical.

In view of this study, over 49 % of areas cities’ grain yield in the NCP is below the multi-year average, which means that close to half of the region still has a lot of room for yield improvement in grain production (Fig. 2). The regions with low yields but high levels of irrigation in southern, the efficiency of irrigation water is very low, so it is urgent to popularize water-saving irrigation technology. We suggested adopting rationed sprinkler irrigation technology in these regions to reduce water wastage by adjusting the irrigation amount according to regional soil moisture and crop water requirement [17,[67], [68], [69]]. Sprinkler irrigation has been widely used for winter wheat crops in the NCP to maintain high crop yield and enhance water use efficiency because water resources are scarce. Moreover, a previous experiment in the NCP revealed that grain yield was greater by 11.5–50.9 % in the sprinkler than in the surface irrigated field [70].

Besides, for the medium-yield areas in the mid-west that show low and negatively correlated levels of irrigation, improving the irrigation infrastructure and adopting deficit irrigation and water-saving irrigation technologies is also necessary [[71], [72], [73]], including expanding existing storage capacity and adopting sprinkler irrigation [74,75], and applying deficit irrigation on the maize at the vegetative growth stage [69]. Moreover, soil moisture management such as straw mulching and other methods to minimize soil moisture evaporation was recommended [76].

In the high grain yield regions of the east, where effective irrigation has plateaued, indicating high irrigation water efficiency approaching the marginal limit, it is recommended to maintain existing irrigation methods to ensure stable grain production. Meanwhile, further exploration of more water-efficient irrigation methods should be pursued to sustain stable food production with lower water consumption, such as precision irrigation technologies [12,28,66,77,78], including smart irrigation systems [79,80] and field micro-irrigation [81]. Implementing appropriate irrigation scheduling that allocates limited water resources in time and space can enhance the marginal benefit per unit of water, striking a balance between water conservation and high crop yield [78].

On the other hand, since the correlation between winter wheat and irrigation is generally weak, we recommend selecting varieties with high water utilization, especially in low and medium yielding regions where the correlation with irrigation is negative. Meanwhile, maize in low-yield regions can also attempt to adopt varieties that are heat resistant and with high water utilization. Much evidence found significant differences in the effects of water-deficit stress across varieties [82]. For example, breeding drought and heat resistant crop varieties can reduce irrigation inputs and are expected to have the capacity to save 4 billion tons of water while increasing yields by 1995 million kilograms (http://ics.caas.cn//xwdt/kyjz/236500.htm). In summary, securing a stable increase in grain production in the NCP will require a combination of agricultural technological advances and crop variety selection rather than simply increasing resource inputs such as irrigation water.

This paper is limited to the objective of achieving the relationship between wheat and maize yields and irrigation. However, other factors that may affect regional agricultural production were not taken into account in this study, such as adjusting sowing dates [83], crop variety improvement [84], soil properties [85], fertilizer application [55,56], climate change [54]. Therefore, future studies could explore the relationship between these factors and crop yield to enhance our comprehension of the quantitative association between food yield and irrigation.

## 4 Conclusion

Driven by the quest to derive rational irrigation strategies from historical coupling patterns of irrigation and grain yields, this study aimed to gain a contextual understanding of the geographical correlation between irrigation and grain yield dynamics.

The NCP case study showed significant variation in grain yield among cities, with higher yields in the south and lower yields in the north. Maize yields were slightly higher than wheat. The correlation between grain yields and irrigation was high in the north and low in the south, consistent with the spatial distribution of grain yield. Though irrigation has barely contributed to yield increases in the NCP since 2002. The correlation between irrigation and maize yield was more significant than that for wheat.

Additionally, there was a noticeable decrease in irrigation across approximately 49 % of the areas since 2004, while effective irrigated areas remained relatively low at over 70 % of the NCP, mainly concentrated in central and western regions. A strong spatial aggregation was observed in the geographical distribution of grain yields and irrigation within the NCP. Irrigation intensity has consistently decreased across most of the NCP and increased only in 16 % of areas.

Irrigation strategies were derived according to the geographical correlation between irrigation and grain yield in the NCP. We recommend the promotion of sprinkler irrigation in the low-yield and low-irrigation correlation regions in the south. In the middle yield with negative irrigation correlation majority regions in the central part, we propose improving the irrigation infrastructure and adopting deficit irrigation and water-saving irrigation technologies. Moreover, maintain current irrigation strategies in high-yield regions.

We contend that the utilization of optimal grain varieties, coupled with adjustments in irrigation techniques, has the potential to mitigate irrigation water usage for sustainable grain production in the NCP. Therefore, we suggest promoting wheat varieties with higher water use efficiency in the southern region, and drought-resistant wheat varieties with higher water utilization in regions where significant grain yield-irrigation negative correlations predominate. Especially, selecting maize varieties with higher water use efficiency is necessary for medium- and low-maize yielding areas.

This study has highlighted the significance of formulating reasonable irrigation strategies through understanding the geographic correlation and dynamics between irrigation and grain yield. We argue that findings of such studies in various regions or globally will hopefully benefit achieving food security while keeping agriculture systems sustainable.

## Data availability

Data included in article/supp. material/referenced in article.

## CRediT authorship contribution statement

**Yulian Gao:** Writing – review & editing, Writing – original draft, Visualization, Validation, Software, Formal analysis. **Yaojie Yue:** Writing – review & editing, Writing – original draft, Supervision, Resources, Project administration, Methodology, Funding acquisition, Formal analysis, Conceptualization. **Wuqiong Yang:** Writing – original draft, Visualization, Software, Resources, Methodology, Investigation, Data curation.

## Declaration of competing interest

The authors declare the following financial interests/personal relationships which may be considered as potential competing interests: Yaojie Yue reports financial support was provided by 10.13039/501100001809National Natural Science Foundation of China. If there are other authors, they declare that they have no known competing financial interests or personal relationships that could have appeared to influence the work reported in this paper.
